# Papel del laboratorio clínico en el estudio de nefrolitiasis y cristaluria por mesalazina

**DOI:** 10.1515/almed-2025-0094

**Published:** 2025-08-11

**Authors:** Ana Isabel Balbuena, Noelia Poveda, Araceli López, María José Ferri

**Affiliations:** Servicio de Análisis Clínicos, Hospital General Universitario Virgen de la Salud, Elda, Alicante, España; Servicio de Análisis Clínicos, Hospital General Universitario Doctor Balmis, Alicante, España; Comisión de Nefrología de la Sociedad Española de Medicina de Laboratorio, Elda (Alicante), España

**Keywords:** cristaluria, litiasis renal, mesalazina

## Abstract

**Objetivos:**

La formación de litiasis urinarias es una complicación relativamente frecuente en los pacientes con enfermedad inflamatoria intestinal (EII). Su origen se debe a varios factores y pueden producir complicaciones de diversa gravedad. Una de las causas es el propio tratamiento farmacológico.

**Caso clínico:**

Presentamos 6 casos de pacientes con EII tratados con mesalazina que presentaron cólicos renoureterales. En 3 de ellos se produjo la expulsión de litiasis, cuyo análisis mediante EIR (espectroscopia infrarroja), confirmó su composición 100 % mesalazina. En los otros 3 casos se objetivó la presencia de cristaluria en el estudio del sedimento urinario durante el CRU. En 2 de estos pacientes, se analizó el sedimento urinario, previamente desecado, mediante EIR, confirmándose su composición por mesalazina.

**Conclusiones:**

Aunque se ha descrito la aparición de litiasis con el uso de este fármaco, se desconoce su frecuencia y existen pocos casos publicados. Respecto a la cristaluria por mesalazina, confirmada en dos de los casos presentados, sólo se había documentado un caso anteriormente, debido a la dificultad en su identificación. Queremos incidir en la importancia del estudio del sedimento urinario en el seguimiento de los pacientes en tratamiento con mesalazina, con el fin de identificar la presencia de cristaluria y evitar así complicaciones mayores.

## Introducción

Los pacientes con enfermedad inflamatoria intestinal presentan un riesgo elevado de presentar litiasis renales, con una incidencia de alrededor del doble que la población sana. En estos pacientes, además, los cálculos renales se asocian con una mayor morbilidad [1]. La patogénesis de los mismos es multifactorial, incluyendo la inflamación y la malabsorción, con las consecuentes alteraciones en el estado de hidratación y equilibrio electrolítico. Es necesario ser conscientes de este mayor riesgo, para establecer un diagnóstico precoz y evitar complicaciones, como infecciones urinarias, sepsis, insuficiencia renal aguda, llegando incluso al desarrollo de nefritis intersticial y enfermedad renal crónica [2], 3]. En estos pacientes los cálculos más frecuentes están constituidos por oxalato cálcico y ácido úrico. Es por ello que en muchos casos en los que presentan cólicos renales, no se piensa en la posible composición farmacológica de los mismos.

El paso inicial para la generación de cálculos renales es la formación de cristales. La cristaluria de origen medicamentoso no es un hallazgo frecuente en el estudio del sedimento urinario, pero puede llegar a originar un fracaso renal agudo. En muchas ocasiones no se llega a identificar la composición de estos cristales. La urolitiasis inducida por fármacos se estima entre el 1–2% en la población general, las causas de su producción pueden ser por efectos metabólicos a nivel renal o en el caso de fármacos poco solubles con alta tasa de excreción renal, a la precipitación del propio fármaco o sus metabolitos en los conductos urinarios [4], [5], [6], [7]. La diferenciación de estos cálculos presenta algunas dificultades. De las diferentes metodologías disponibles para su estudio, el análisis químico no permite identificar las litiasis medicamentosas y las técnicas de imagen (radiografía, TAC, ecografía), aunque pueden orientar a su composición por las características morfológicas, no permiten conocer su composición exacta. La espectroscopia infrarroja (EIR) es la técnica recomendada por la Asociación Europea de Urología. Esta técnica permite la identificación exacta de los fármacos litógenos al comparar su espectro con espectros ya conocidos, pero no está disponible en todos los hospitales. Ha de tenerse en cuenta además que si el espectro no se encuentra en la base de datos pueden obtenerse resultados no concluyentes, lo que puede contribuir a un infradiagnóstico de los mismos.

La mesalazina (ácido 5-aminosalicílico, 5-ASA) es un fármaco antiinflamatorio utilizado en primera línea del tratamiento de la enfermedad inflamatoria intestinal. Se usa por vía oral o por vía rectal y la dosis recomendada en adultos es de 2 g/día. Administrada por vía oral se absorbe en el intestino en un 60 %, posteriormente es metabolizada en el hígado y otros tejidos y se excreta principalmente por el riñón. Como reacciones adversas asociadas a su uso, la agencia española de medicamentos y productos sanitarios (AEMPS) hace constar en su ficha técnica pericarditis, pancreatitis, hepatitis, nefrotoxicidad, alveolitis, discrasias sanguíneas y alteración de la función renal, como nefritis intersticial aguda y crónica, síndrome nefrótico e insuficiencia renal, con una frecuencia muy rara (inferior a 1:10.000) y, con una frecuencia no conocida o que no se puede estimar, nefrolitiasis y decoloración de la orina [8]. Se ha descrito la presentación de cólicos nefríticos como efecto adverso con su uso y existen algunos casos publicados de nefrolitiasis medicamentosa por mesalazina [1], 4], 5] constatando en sólo uno de ellos la presencia de cristaluria [4]. También se ha publicado un caso de hematuria como reacción adversa medicamentosa a mesalazina [9].

## Caso clínico

Presentamos 3 casos de litiasis renales y 3 casos de cristaluria en pacientes con EII tratados con mesalazina que han presentado uno o más episodios de cólico renoureteral (CRU) como complicación. El análisis de la composición de los cálculos y de los cristales del sedimento urinario se realizó mediante EIR (Spectrum Two; Perkin Elmer; (Waltham, Massachusetts, Estados Unidos)) en 5 de los 6 casos.

**Figura 1:**
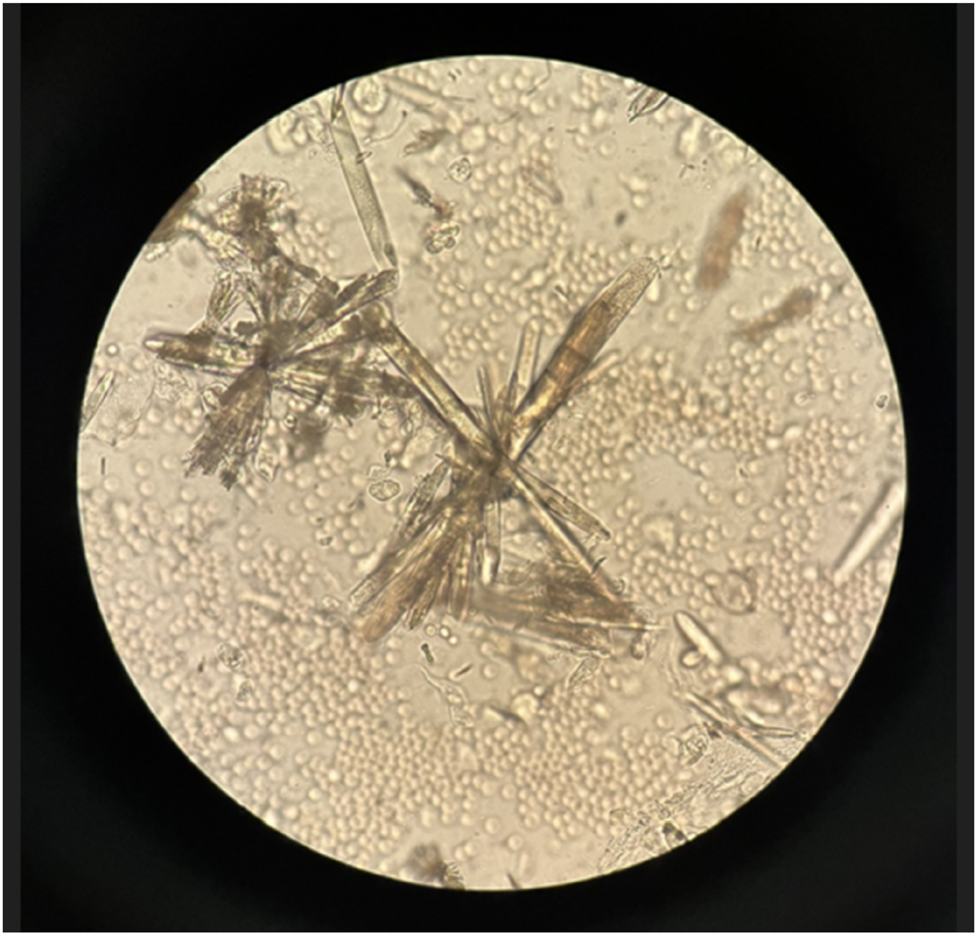
Cristales de mesalazina en sedimento urinario. Observación en campo claro a 400× en microscopio óptico Olympus CX41.

De los 6 casos presentados, 5 tenían como diagnóstico colitis ulcerosa y uno enfermedad de Crohn (Tabla 1). Se trata de 3 hombres y 3 mujeres, con edades comprendidas entre los 21 y los 43 años. Todos ellos tenían en común estar en tratamiento con mesalazina por vía oral a una dosis de 4 gramos al día y haber presentado al menos un episodio de CRU entre mayo de 2022 y octubre de 2024. La mayoría presentado presentaron múltiples episodios de cólicos renoureterales sin existir antecedentes previos documentados de los mismos antes de iniciar el tratamiento. En 2 de los pacientes se han documentado más de 10 episodios y en otro, aunque sólo hay 5 episodios documentados, en su historia clínica se hace referencia a más de 10 en un año. Respecto al tiempo transcurrido desde el comienzo del tratamiento con mesalazina hasta la aparición del primer cólico renoureteral documentado, es muy variable, desde menos de un mes en 2 de los casos hasta 8 años en otro. Hubo 2 pacientes que presentaron hidronefrosis como complicación durante un episodio de CRU. En 3 de los 6 casos se produjo la expulsión de litiasis cuyo análisis mediante EIR confirmó que su composición era 100 % mesalazina. En los otros 3 casos no hubo expulsión de cálculo pero sí se objetivó la presencia de cristaluria en el estudio microscópico del sedimento urinario coincidiendo con el episodio de CRU (Figuras 1 y 2). En el primer caso observado no se pudo identificar la composición de los cristales. En los otros 2 casos se procedió al análisis del sedimento urinario, previamente desecado, mediante EIR, confirmándose su composición por mesalazina (Figura 3). En 2 de los pacientes que presentaron litiasis se suspendió el tratamiento oral con mesalazina y en uno de los que presentaron cristaluria se disminuyó la dosis a 2 g/día. En un caso se produjo el abandono del tratamiento por parte del propio paciente.

**Tabla 1:** Presencia de litiasis/cristaluria en pacientes con EII tratados con mesalazina que han presentado uno o más episodios de CRU.

Caso	1	2	3	4	5	6
Sexo	Mujer	Mujer	Hombre	Mujer	Hombre	Hombre
Edad	21	42	43	38	25	32
Diagnóstico	Enfermedad de Crohn	Colitis ulcerosa	Colitis ulcerosa	Colitis ulcerosa	Colitis ulcerosa	Colitis ulcerosa
Dosis mesalazina	4 g/día	4 g/día	4 g/día	4 g/día	4 g/día	4 g/día
Tiempo de tratamiento	5 años	6 años	4 años	11 años	4 meses	10 años
Episodios de C.R.U.	13	1	4	5	1	12
Tiempo desde inicio del tratamiento hasta primer CRU	<1 mes	5 años	7 meses	3 años	< 1 mes	8 años
Litiasis	No	Sí	Sí	No	No	Sí
Cristaluria	Sí	No	No	Sí	Sí	No
EIR	No	Sí	Sí	Sí	Sí	Sí

CRU, cólico renoureteral; EIR, espectroscopia infrarroja.

**Figura 2:**
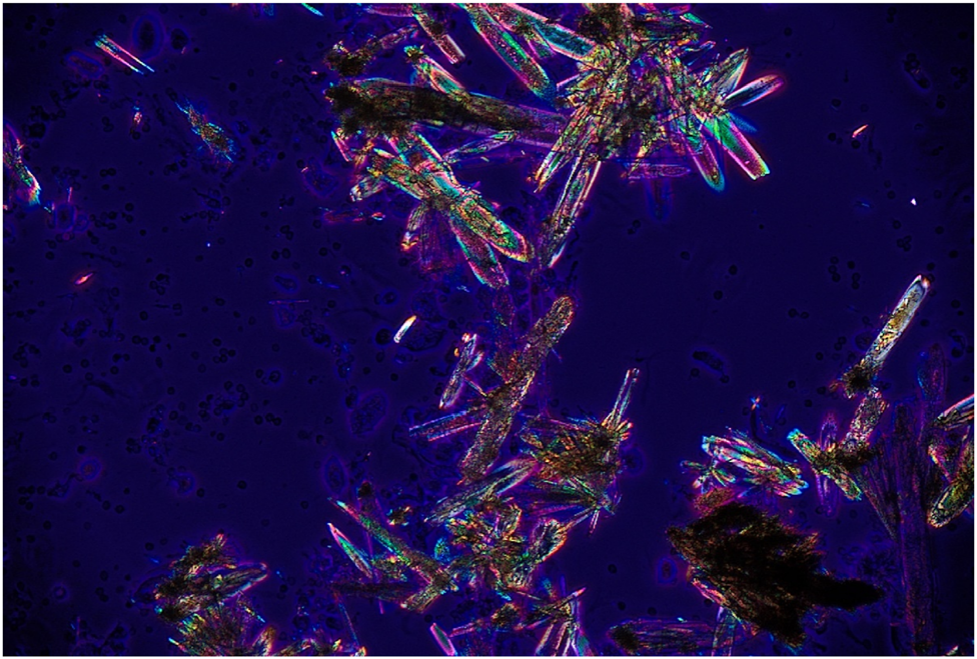
Cristales de mesalazina en sedimento urinario. Observación con luz polarizada a 400× en microscopio Olympus CX43.

**Figura 3:**
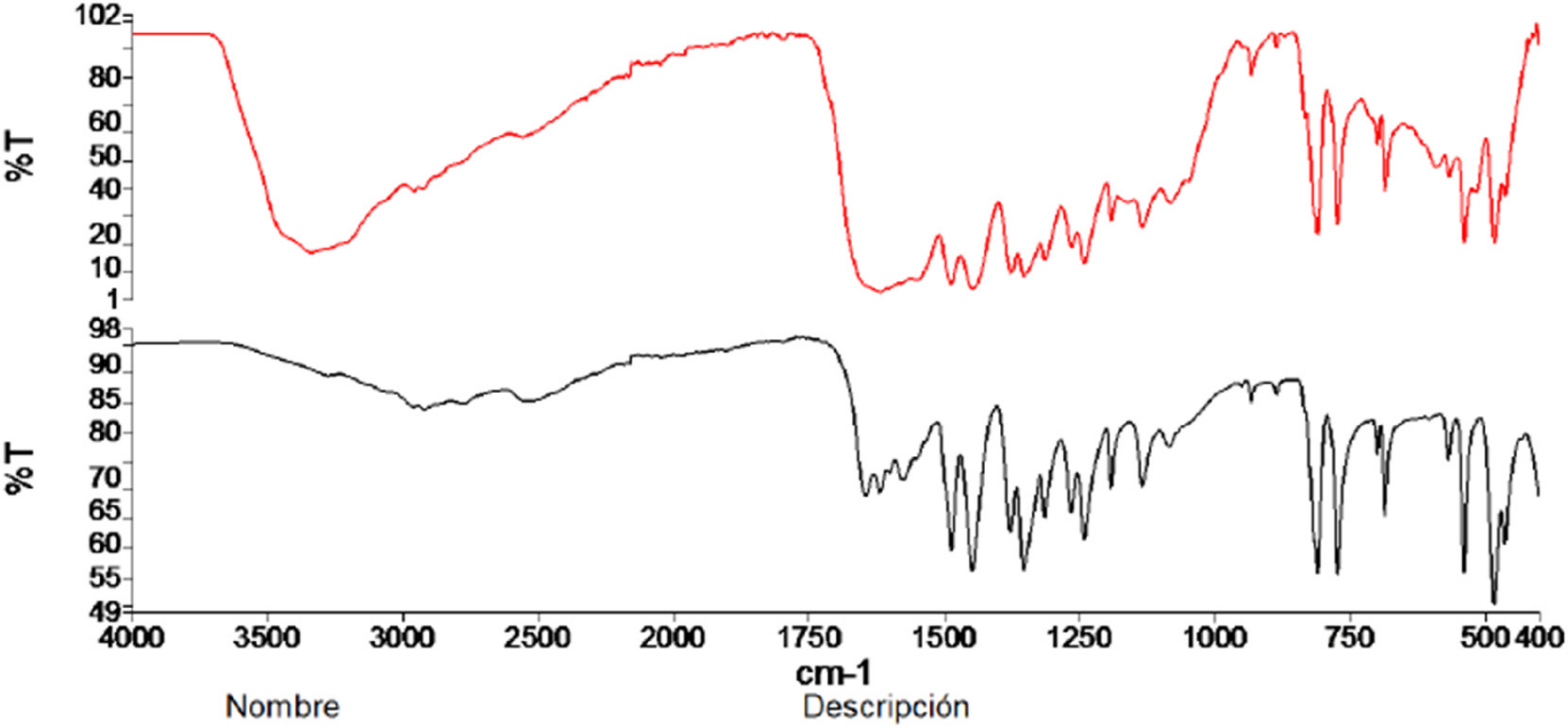
Imagen de espectroscopia infrarroja de sedimento urinario con cristaluria por mesalazina. En negro espectro teórico, en rojo espectro del paciente.

## Discusión

La nefrolitiasis por mesalazina es una reacción adversa descrita por la AEMPS, aunque hasta el momento actual no se ha podido establecer la frecuencia de aparición de la misma. La mayoría de los casos se han reportado en los últimos 3 años, entre el primer y sexto mes de tratamiento, dándose por igual en hombres y en mujeres [10]. Existen pocas publicaciones con series de casos en los que se ha podido confirmar la composición de las litiasis. Respecto a la presencia de cristaluria por mesalazina, aparte de los aquí expuestos, sólo se hace mención en un caso de la serie publicada por *Chebion* et al. en 2021 [4].

La cristaluria es el paso previo a la litiasis. Se ha demostrado que la presencia de cristales de gran tamaño, los agregados y la cristaluria persistente refleja con precisión una propensión para la formación de cálculos [11]. En las cristalurias de origen medicamentoso no siempre se identifica la composición de los cristales al no disponer de la metodología necesaria para ello. Además pueden pasar desapercibidas si el paciente no presenta síntomas urinarios y/o alguna alteración en los parámetros de la tira reactiva de orina que lleven al estudio del sedimento al microscopio óptico. En los pacientes en tratamiento con mesalazina la observación de cristales en el sedimento urinario es un hallazgo de especial interés, ya que puede ser indicativo del riesgo litogénico. Sin embargo, el volumen de muestras de orina analizadas a diario y el no disponer de información clínica en la mayoría de los casos, hace que se puedan pasar por alto. En el momento actual, en el trabajo de rutina del laboratorio no se dispone de un procedimiento automatizado para la detección de cristales no habituales en orina, sino que constituyen un hallazgo cuando se estudia el sedimento por otros motivos, o se solicita su estudio explícitamente.

## Puntos de aprendizaje

– El tratamiento con mesalazina se puede asociar con frecuencia a cólicos renoureterales con la presencia de cristaluria así como la formación de cálculos urinarios.
– El estudio del sedimento urinario en estos pacientes es una herramienta valiosa para el diagnóstico etiológico de estos cólicos renoureterales.
– En el seguimiento de estos pacientes sería conveniente realizar estudio del sedimento urinario para detectar con anticipación la aparición de estas complicaciones, dando la oportunidad al clínico de modificar el tratamiento o la posología del fármaco.
– Es fundamental la comunicación del clínico con el especialista de laboratorio ante la sospecha diagnóstica, facilitando datos clínicos y solicitando el estudio del sedimento urinario dirigido a la búsqueda de estos cristales.
